# ACE2-Mediated Reduction of Oxidative Stress in the Central Nervous System Is Associated with Improvement of Autonomic Function

**DOI:** 10.1371/journal.pone.0022682

**Published:** 2011-07-27

**Authors:** Huijing Xia, Sonia Suda, Sharell Bindom, Yumei Feng, Susan B. Gurley, Dale Seth, L. Gabriel Navar, Eric Lazartigues

**Affiliations:** 1 Department of Pharmacology & Experimental Therapeutics and Cardiovascular Center of Excellence, Louisiana State University Health Sciences Center, New Orleans, Louisiana, United States of America; 2 Department of Physiology Hypertension and Renal Center of Excellence, Tulane University School of Medicine, New Orleans, Louisiana, United States of America; 3 Division of Nephrology, Department of Medicine, Duke University School of Medicine, Durham, North Carolina, United States of America; Roswell Park Cancer Institute, United States of America

## Abstract

Oxidative stress in the central nervous system mediates the increase in sympathetic tone that precedes the development of hypertension. We hypothesized that by transforming Angiotensin-II (AngII) into Ang-(1–7), ACE2 might reduce AngII-mediated oxidative stress in the brain and prevent autonomic dysfunction. To test this hypothesis, a relationship between ACE2 and oxidative stress was first confirmed in a mouse neuroblastoma cell line (Neuro2A cells) treated with AngII and infected with Ad-hACE2. ACE2 overexpression resulted in a reduction of reactive oxygen species (ROS) formation. In vivo, ACE2 knockout (ACE2^−/y^) mice and non-transgenic (NT) littermates were infused with AngII (10 days) and infected with Ad-hACE2 in the paraventricular nucleus (PVN). Baseline blood pressure (BP), AngII and brain ROS levels were not different between young mice (12 weeks). However, cardiac sympathetic tone, brain NADPH oxidase and SOD activities were significantly increased in ACE2^−/y^. Post infusion, plasma and brain AngII levels were also significantly higher in ACE2^−/y^, although BP was similarly increased in both genotypes. ROS formation in the PVN and RVLM was significantly higher in ACE2^−/y^ mice following AngII infusion. Similar phenotypes, i.e. increased oxidative stress, exacerbated dysautonomia and hypertension, were also observed on baseline in mature ACE2^−/y^ mice (48 weeks). ACE2 gene therapy to the PVN reduced AngII-mediated increase in NADPH oxidase activity and normalized cardiac dysautonomia in ACE2^−/y^ mice. Altogether, these data indicate that ACE2 gene deletion promotes age-dependent oxidative stress, autonomic dysfunction and hypertension, while PVN-targeted ACE2 gene therapy decreases ROS formation via NADPH oxidase inhibition and improves autonomic function. Accordingly, ACE2 could represent a new target for the treatment of hypertension-associated dysautonomia and oxidative stress.

## Introduction

Oxidative stress is a condition of increased production of reactive oxygen species (ROS) and/or reduction of scavenging mechanisms. It has been well established that ROS play an important role in the development of autonomic dysfunction preceding cardiovascular diseases, including hypertension [1]. Angiotensin (Ang)-II, one of the major actors of the renin-angiotensin-system (RAS), is a potent activator of NAD(P)H oxidase and is involved in the generation of ROS such as superoxide and hydrogen peroxide [2]. Abundant evidence suggests that a key mechanism by which AngII influences autonomic dysfunction is via its ability to produce ROS [2], [3]. In the past several years, new evidence demonstrated the crucial role of brain ROS in mediating AngII central effects and BP regulation [4], [5], [6]. It has been shown that production of ROS in specific brain regions, including hypothalamus (HT) and rostral ventrolateral medulla (RVLM), mediates sympatho-excitation, leading to hypertension [7], [8]. AngII-mediated ROS generation in the brain also results in impaired baroreflex sensitivity [9], contributing to the increased sympathetic outflow and dysautonomia. Chronic systemic infusion of sub-pressor doses of AngII increases ROS production in the subfornical organ (SFO) and leads to the development of hypertension [4], which is reliant on a Rac1-dependent NAD(P)H oxidase activation [10]. Similarly, acute pressor response to central AngII has been shown to be mediated by NAD(P)H oxidase-dependent production of ROS in hypothalamic cardiovascular regulatory nuclei, including SFO and paraventricular nucleus (PVN) [5]. Taken together, these data support the proposal that ROS are important mediators in RAS-mediated activation of sympathetic tone leading to dysautonomia.

Angiotensin Converting Enzyme 2 (ACE2), an ACE homologue [11], [12], degrades AngII into Ang-(1–7). The presence of ACE2 in brain regions involved in the central regulation of cardiovascular function [13], suggests that ACE2 is part of the brain RAS. Indeed, evidence show that ACE2 interacts with RAS components in the central nervous system (CNS) [14], [15], [16]. Overexpression of ACE2 in the brain decreases blood pressure (BP) and restores autonomic function in hypertensive animals [16], [17] and prevents the AngII-mediated development of hypertension [14], [18]. However, whether ROS are involved in this process is unknown.

It was reported that ACE2 gene deletion leads to an age-dependent cardiomyopathy, which is related to increased AngII-mediated oxidative stress [19]. In addition, ACE2 inhibition exacerbates AngII-mediated ROS production in kidneys [20], while ACE2 gene transfer suppresses AngII-induced increase in NAD(P)H oxidase subunit p22phox expression and lipid peroxidation in a human endothelial cell line [21]. These data suggest that ACE2 may benefit cardiovascular function by opposing AngII-induced oxidative stress in the brain.

In this study, we hypothesized that ACE2 might prevent oxidative stress in the CNS that could be beneficial to regulate sympathetic drive and cardiovascular function. To test this hypothesis, we 1) confirmed the existence of a link between ACE2 and oxidative stress in vitro, 2) determined the impact of ACE2 deletion on ROS formation and autonomic function in vivo, and 3) assessed the potential of brain ACE2 gene therapy in the prevention of oxidative stress and dysautonomia. Our data show that ACE2 deletion resulted in age-dependent increases in oxidative stress, autonomic dysfunction and BP. Moreover, lack of ACE2 exacerbated AngII-mediated oxidative stress in the CNS. Finally, ACE2 gene therapy to the PVN prevented AngII-mediated ROS production in the brain, and restored autonomic function.

## Materials and Methods

### Cell Culture

Neuro2A mouse neuroblastoma cells (ATCC Manassas, VA) were grown in minimum essential medium (MEM; GIBCO®, Invitrogen, Carlsbad, CA) with 2 mM L-glutamine and Earle's balanced salt solution adjusted to contain 1.5 g/L sodium bicarbonate, 0.1 mM non-essential amino acids, 1.0 mM sodium pyruvate and 10% fetal bovine serum (FBS; GIBCO®) at 37°C under a humidified 95% and 5% (v/v) mixture of air and CO_2_. Cells were grown onto chamber slides at a density of 1×10^5^ cells/chamber for dihydroethidine (DHE) fluorescence measurement, or 10 cm dishes at a density of 10^6^ cells/dish for western blot experiments. After 24 hr, cells were used for the following experiments. Each set of experiments was performed in triplicate.

### Western blot

Cells were treated with either vehicle or AngII (100 nmol/L in PBS) for 24 hours, washed with PBS, and collected for western blotting.

Cell pellets were incubated in 300 µL lysis buffer (in mmol/L: HEPES: 10, NaCl: 150, MgCl_2_: 5, EGTA: 1, 0.02% (w/v) NaN_3_, pH 7.4) containing a protease inhibitors cocktail (Sigma, St Louis, MO). The lysate was centrifuged twice at 4,000 rpm, 4°C, for 20 min and the supernatant transferred to a clean tube. Protein concentration was measured using a BCA assay kit (Pearce, Rockford, IL). Cell lysates (30 µg) were mixed with SDS-PAGE sample buffer (0.125 M Tris-HCl, pH 6.8, 4% SDS, 20% glycerol, 10% 2-mercaptoethanol, 0.004% bromophenol blue), heated at 100°C for 5 min and loaded onto a 4–15% SDS–polyacrylamide gel for electrophoresis. Proteins were transferred to nitrocellulose membrane at 200 mA for 3 hr by semi-dry blot (Fisher Scientific, Houston, TX). Membranes were blocked with 5% non-fat milk in PBST (1.47 mM NaH_2_PO_4_, 8.09 mM Na_2_HPO_4_, 145 mM NaCl, 0.05% (v/v) Tween-20®, 0.01% (w/v) thimerosal, pH 7.4) for 1 hr at room temperature and incubated with rabbit anti-mouse ACE2 (Open Biosciences; 1∶500), rabbit anti-AT1R (Santa Cruz, sc-1173; 1∶500), or rabbit-anti-AT2R (Santa Cruz, sc-9040; 1∶500) antibodies, at 4°C, overnight. Membranes were washed with PBST 4 times for 5 min then incubated with goat anti-rabbit IgG-HRP (Perkin Elmer; 1∶5000) for 1 hour at room temperature. Specific bands were detected by chemiluminescence according to the manufacturer's instructions (ECL®, Perkin Elmer) for 15 minutes at room temperature and re-probed with mouse anti-α-tubulin (Abcam, ab7291; 1∶10000) and goat anti-mouse IgG-HRP. Bands were quantified by laser densitometry (FujiFilm, ImageReader version 1.2). Bands corresponding to specific antibodies were normalized to α-tubulin.

### Transgenic mice and animal husbandry

Experiments were performed in young adult (12 weeks age) and mature (48 weeks age) male ACE2 knockout (ACE2^−/y^) mice (C57bl/6J background) [22] and their non-transgenic (NT) littermates. Animals were fed standard mouse chow and water *ad libitum*. All procedures were approved by the LSU Health Sciences Center Animal Care and Use Committee and are in agreement with the National Institutes of Health Guide for the Care and Use of Laboratory Animals (IACUC #2703).

### Physiological recordings

Baseline BP was measured in ACE2^−/y^ and NT mice during 3 days using radio-telemetry, as described [16]. Young mice were then infused subcutaneously with AngII (600 ng/kg/min), or vehicle (0.9% saline) for 10 days using osmotic pumps (Alzet). BP was continually recorded during infusion.

Autonomic function was assessed before and after AngII infusion using a pharmacological method involving ip injection of propranolol (β-blocker, 4 mg/kg), atropine (muscarinic receptor blocker, 1 mg/kg) and chlorisondamine (ganglionic blocker, 10 mg/kg). Changes in HR (ΔHR) and BP (ΔBP) were calculated following administration of these blockers [16].

In another set of experiments, young ACE2^−/y^ mice were infused subcutaneously with AngII (600 ng/kg/min), or vehicle (0.9% saline) for 10 days. On the 3^rd^ day of AngII infusion, mice were anesthetized with sodium pentobarbital (50 mg/kg, ip) and placed in a stereotaxic frame for injection with Ad-hACE2-eGFP or Ad-eGFP. A glass micropipette connected to a pressure injector (UMP2, WPI) and containing the virus was lowered into the brain for bilateral PVN injection (4×10^6^ pfu/200 nl/side) using the following coordinates (0.3 mm lateral, 0.5 mm caudal, 5.0 mm ventral). Following injection, mice were returned to their home cage and cardiovascular parameters recorded continuously.

At the end of AngII infusion, mice were sacrificed and brain regions harvested for the following assays.

### Fluorogenic Monitoring of Superoxide Production

Neuro2A cells were incubated in serum free medium (SFM) in presence of Ad-hACE2-eGFP or Ad-eGFP control virus (100 MOI) for 48 hr [14]. On the third day post infection, GFP expression was observed, using a fluorescence microscope (Olympus, IX81; Excitation/emission wavelengths: 488/509 nm), as an index of successful infection and cells treated for 30 minutes with either vehicle, AngII (100 nmol/L) alone or losartan (1 µmol/L)+AngII. The AT1R antagonist Losartan was administered 30 min prior AngII. The oxidant-sensitive fluorogenic probe DHE (2 µmol/L) was loaded at the same time of AngII. Each experiment was performed in triplicate. Cells were washed thrice in the dark with PBS and examined on a fluorescence microscope (Olympus U-TB190, Japan; Excitation/Emission wavelength: 518/605 nm).

Mouse brains were rapidly dissected, frozen in OCT and stored at −80°C until use. Brains were cryostat-sectioned to 30 µm and directly mounted onto chilled microscope slides. Sections were thawed at room temperature, rehydrated with PBS, incubated with DHE (1 µM in PBS) for 5 min in the dark. After incubation, sections were washed thrice for 3 min with PBS. Following the final wash, sections were coversliped and imaged using a fluorescence microscope (Olympus U-TB190, Japan; Excitation/Emission wavelengths: 518/605 nm).

DHE fluorescence was quantified using Image J.

### AngII measurement

AngII levels in the mouse plasma and brain were measured by radioimmunoassay (RIA) as previously reported [23], [24]. Briefly, blood and brain samples were harvested following decapitation. Trunk blood was collected in chilled tubes containing a mixed inhibitor solution, microfuged at room temperature, then the plasma was transferred to ice cold methanol (100%). Brain samples were immersed in cold methanol (100%) and homogenized immediately upon harvesting. Plasma and brain homogenates were centrifuged and the supernatants were dried overnight in a vacuum centrifuge. The dried fraction was resuspended in RIA buffer and AngII levels quantitated.

### ACE2 activity assay

ACE2 activity was measured in the hypothalamus and ventral lateral medulla (VLM), as previously reported [16].

### NADPH oxidase and SOD activity assay

HT and VLM were homogenized in homogenization buffer (in mmol/L: HEPES: 20, EDTA: 1) containing a protease inhibitors cocktail (Sigma, St Louis, MO). The lysate was centrifuged twice at 3,000 rpm, 4°C for 15 min and the supernatant transferred to a clean tube. Protein concentration was measured using a BCA assay kit (Pearce, Rockford, IL).

NADPH oxidase activity was measured by a luminescence assay. Experiments were performed on a microplate luminometer (1420 Luminescence Counter, Perkin Elmer) at 37°C using 100 µg protein/well, NADPH 200 µmol/L and lucigenin 10 µmol/L in 200 µl reaction buffer (in mmol/L: HEPES: 20, NaCl: 9.9, KCl: 4.7, MgSO4: 1.2, KH2PO4: 1, CaCl2: 1.9, NaHCO3: 25, glucose: 11.1, pH 7.4). In some experiments, oxidase activity was measured in the presence of a flavoprotein inhibitor, diphenyleneiodonium (DPI, 10 µmol/L), to confirm that the measured activity was attributable to NADPH oxidase. A buffer blank (<2% of the homogenate signal) was subtracted from each reading. Chemiluminescence readings were obtained at 60 sec intervals for an overall measuring time of 40 min, a period over which maximal chemiluminescence was achieved. Luminescence units (AU) were normalized to protein amount.

SOD activity in brain homogenates was measured with the SOD Assay kit (Applied Bioanalytical Labs) according to the manufacturer's protocol. The absorbance units (AU) were normalized to protein amount.

### Statistical Analysis

Data are expressed as mean ±SEM. Data were analyzed, when appropriate, by Student's *t* test, repeated measures ANOVA or one-way ANOVA (after Bartlett test of homogeneity of variance) followed by Tukey-Kramer correction for multiple comparisons between means. Statistical comparisons were performed using Prism5 (GraphPad Software, San Diego, CA). Differences were considered statistically significant at *P*<0.05.

## Results

### ACE2 reduces AngII-mediated oxidative stress in Neuro2A cells

**Figure 1:**
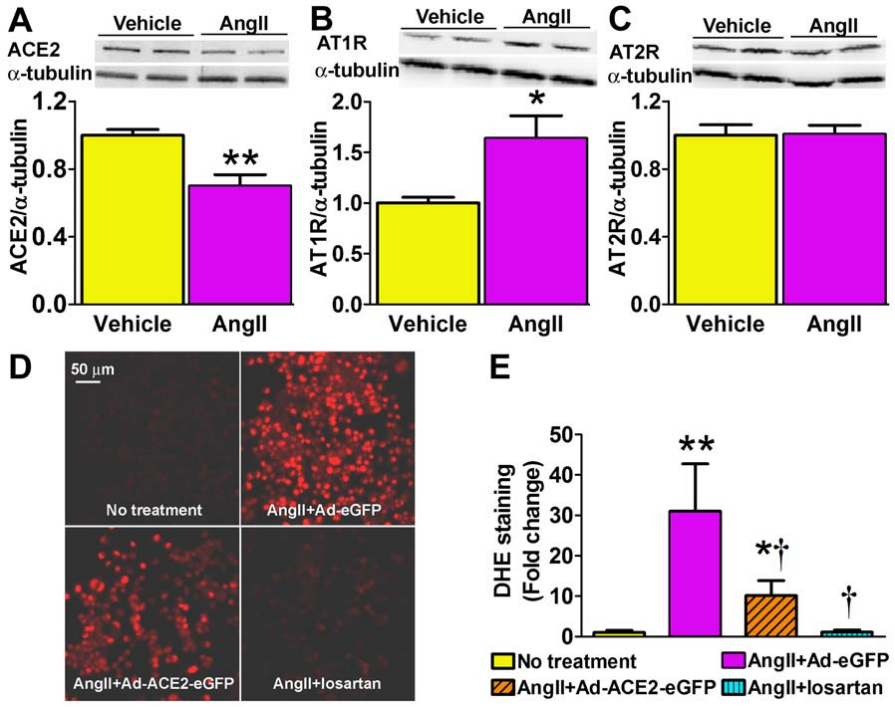
Effects of AngII and ACE2 on oxidative stress in Neuro2A cells. Western blots showed that AngII treatment (100 nmol/L, 24 h) decreased ACE2 (A; 100 KDa, n = 6, **P<0.01 vs. vehicle), increased AT1R (43 KDa; B; n = 6, *P<0.05 vs. vehicle) without changing AT2R (44 KDa; C; *P*>0.05 vs. vehicle) protein expression. Representative DHE staining (D) and quantified data (E), showing that AngII (100 nmol/L, 30 min) significantly increased ROS production (n = 6, **P<0.01 vs. no treatment). Pre-treatment with Ad-ACE2-eGFP reduced AngII-stimulated ROS formation (n = 6, †*P*<0.05 vs. AngII+Ad-eGFP, *P<0.05 vs. no treatment). Blockade of AT1R with losartan completely prevented AngII-mediated ROS production (n = 6, †*P*<0.05 vs. AngII+Ad-eGFP).

The relationship between ACE2 and AngII-mediated oxidative stress in neurons was first assessed using an *in vitro* approach. Western bloting showed that Neuro2A cells treated with AngII exhibited a significant reduction in ACE2 expression compared to vehicle treatment (0.70±0.06 vs. 1.0±0.03, *P*<0.01; Figure 1A). This decrease was accompanied by increased AT1R expression (1.64±0.22 vs. 1.0±0.06, *P*<0.05; Figure 1B) while the AT2R level was not affected (1.01±0.05 vs. 1.0±0.06, *P*>0.05; Figure 1C).

To determine whether ACE2 could be beneficial in modulating AngII-mediated ROS formation, cells were infected with Ad-hACE2 virus and oxidative stress assessed by DHE staining. AngII treatment significantly increased ROS in Neuro2A cells (31±11 vs. 1.0±0.5 fold, *P*<0.01 vs. no treatment; Figure 1D,E), while pre-infection with Ad-ACE2 significantly attenuated the AngII-induced oxidative stress (10.1±3.7 fold, *P*<0.05; Figure 1D,E). Losartan pre-treatment completely prevented the AngII-mediated ROS generation (1.1±0.5 fold, *P*<0.05 vs. AngII; Figure 1D,E). Taken together, these data support the beneficial role of ACE2 in modulating AngII-mediated ROS formation in neurons.

### Impaired cardiac sympathetic and vagal tone in ACE2^−/y^ mice

**Figure 2:**
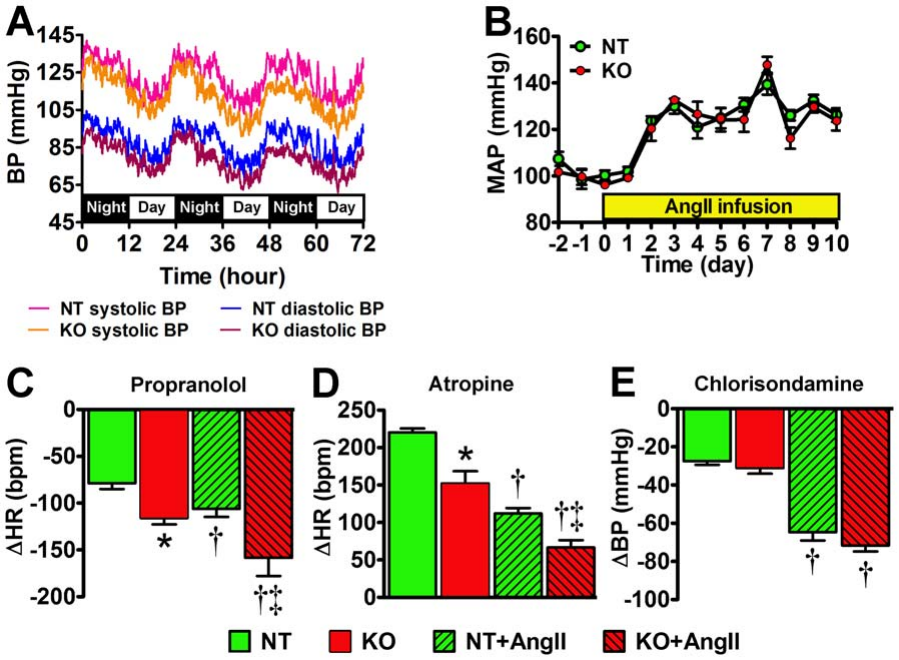
Normal blood pressure and increased autonomic dysfunction in young ACE2^−/y^ mice. Baseline blood pressure traces from young non-transgenic (NT) and ACE2^−/y^ (KO) mice over 3 days recording show no differences between genotypes (A). Mean arterial pressure (MAP) was similarly increased in both NT and KO mice during AngII infusion (B). KO mice exhibited enhanced bradycardia to propranolol (C; *P<0.05 vs. NT; n = 8) and reduced tachycardia following atropine (D; *P<0.05 vs. NT; n = 8). AngII infusion significantly increased sympathetic drive (C) and reduced vagal tone (D) in both NT and KO (†*P*<0.05 vs. baseline, n = 8). Autonomic dysfunction remained more pronounced in KO compared to NT following AngII infusion. (C,D; ‡*P*<0.05 vs. NT+AngII). Changes in BP following ganglionic blockade, an index of vascular sympathetic drive, were not different between NT and KO on baseline or following AngII infusion (E; *P*>0.05).

To determine the role of ACE2 *in vivo*, we first investigated the consequences of ACE2 gene deletion on cardiovascular function. We measured BP and autonomic function in young adult (12 weeks) ACE2^−/y^ mice, on baseline and during AngII infusion. Baseline BP was not different between ACE2^−/y^ and NT over 3 days recording [mean arterial pressure (MAP): 100±1 vs. 102±3 mmHg, *P*>0.05; Figure 2A,B]. AngII infusion (10 days) similarly increased MAP in both ACE2^−/y^ and NT mice (124±4 vs. 126±3 mmHg, *P*>0.05; Figure 2B). Baseline cardiac sympathetic drive was significantly higher and vagal tone blunted in ACE2^−/y^ compared to NT mice (*P*<0.05; Figure 2C,D). However, vascular sympathetic drive was not altered in ACE2^−/y^ (*P*>0.05; Figure 2E). AngII infusion exacerbated cardiac dysautonomia in all mice but these modifications were more pronounced in ACE2^−/y^ (*P*<0.05; Figure 2C,D). Vascular sympathetic drive was similarly increased in both groups. Altogether these data suggest that young ACE2^−/y^ mice exhibit basal cardiac dysautonomia but not hypertension.

### ACE2 deletion exacerbates AngII-mediated oxidative stress in young mice

**Figure 3:**
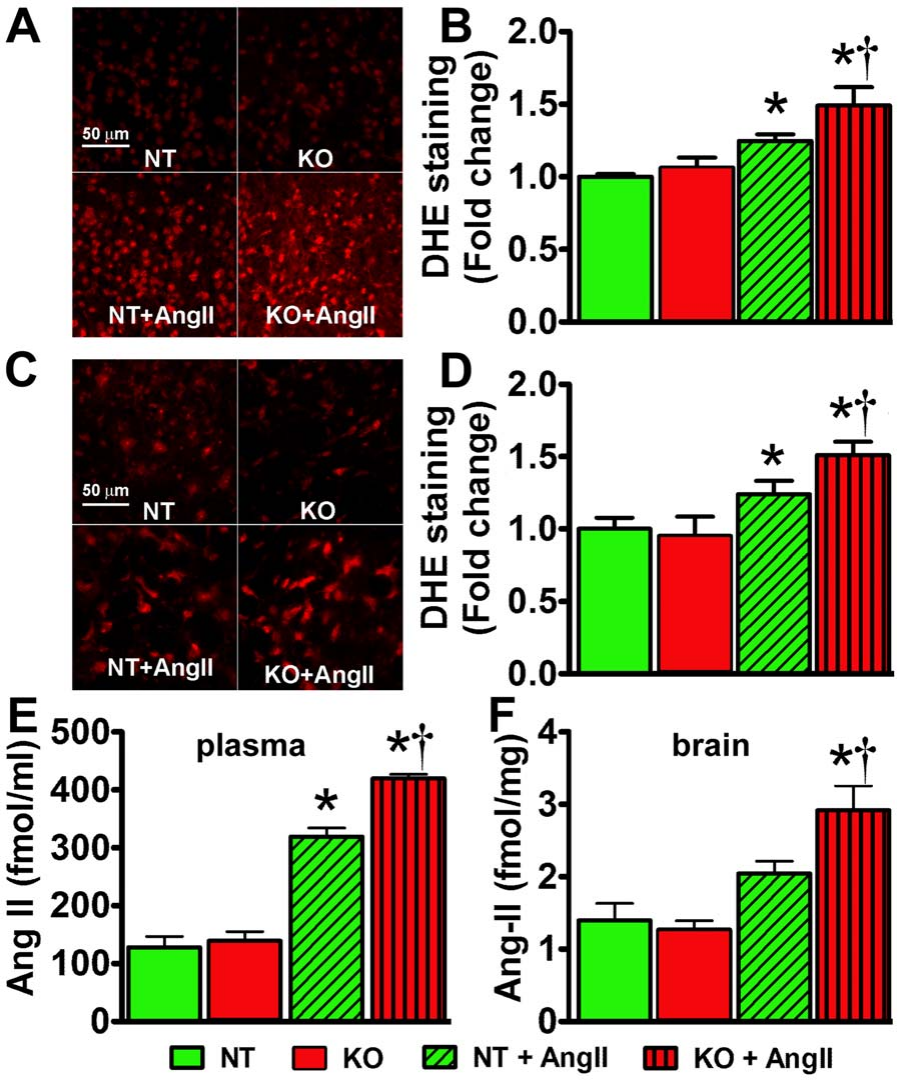
ROS and AngII levels in young ACE2^−/y^ mice. Representative DHE staining and quantified data showing no difference in baseline ROS levels in the PVN (A, B) or RVLM (C, D) between KO and NT mice (*P*>0.05, n = 8). AngII infusion significantly increased ROS production in these regions in both genotypes (A–D; *P<0.05 vs. baseline, n = 8), with higher levels in KO (A–D; †*P*<0.05 vs. NT+AngII). Baseline AngII levels in both plasma (E) and brain (F) were not different between mice (*P*>0.05, n = 8). While AngII infusion significantly increased plasma (E) AngII levels in both genotypes, only KO mice show elevated brain AngII levels (F) (*P<0.05 vs. baseline, n = 8). KO mice exhibited higher plasma and central AngII levels following AngII infusion (†*P*<0.05, vs. NT+AngII).

**Figure 4:**
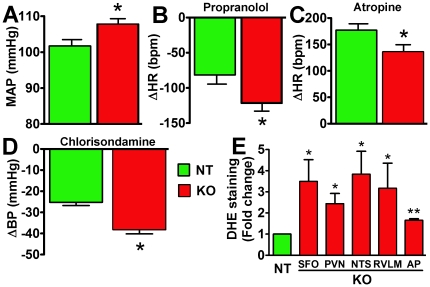
Increased BP, autonomic dysfunction and oxidative stress in mature ACE2^−/y^ mice. Baseline MAP was significantly increased in mature ACE2^−/y^ compared to age-matched NT mice (A). Increased sympathetic (B) and decreased parasympathetic (C) tones were also observed in ACE2^−/y^ mice (*P<0.05 vs. NT). In addition, vascular sympathetic tone in ACE2^−/y^ mice was significantly elevated compared to NT (D; *P<0.05 vs. NT). Moreover, oxidative stress was exacerbated in the several brain regions (SFO: subfornical organ, PVN: paraventricular ncleus, NTS: nucleus of the tractus solitarius, RVLM: rostral ventrolateral medulla and AP: area postrema) of mature ACE2^−/y^ compared to NT mice (E; *P<0.05 vs. NT).

To investigate whether ACE2 modulates AngII-mediated oxidative stress in vivo, we measured ROS and AngII levels in the brain of ACE2^−/y^ mice. DHE staining showed that ROS levels were not different in the PVN and RVLM of young ACE2^−/y^ on baseline compared to NT (*P*>0.05; Figure 3A–D). However, ROS were significantly more elevated in the PVN (1.49±0.12 vs. 1.25±0.04, *P*<0.05; Figure 3A,B), RVLM (1.51±0.09 vs. 1.24±0.09, *P*<0.05; Figure 3C,D) and in other brain regions (data not shown) of young ACE2^−/y^ following AngII infusion. Baseline AngII levels were not different in the plasma (140±16 vs. 128±19 fmol/ml, *P*>0.05; Figure 3E) or brain (1.3±0.1 vs. 1.4±0.2 fmol/mg, *P*>0.05; Figure 3F) of ACE2^−/y^ compared to NT. In NT mice, AngII infusion significantly increased AngII in the plasma (Figure 3E) but not in the brain (Figure 4F). However, ACE2^−/y^ mice exhibited higher AngII levels than NT in both plasma (420±7 vs. 319±15 fmol/ml, *P*<0.05; Figure 3E) and brain (2.91±0.34 vs. 2.05±0.17 fmol/mg, *P*<0.05; Figure 3F). These data suggest that lack of ACE2 leads to impaired AngII metabolism, thus resulting in exacerbation of oxidative stress.

### Increased BP, dysautonomia and oxidative stress in mature ACE2^−/y^ mice

Because previous work suggested that ACE2 gene deletion leads to age-dependent cardiomyopathy [19], we investigated BP regulation and oxidative stress in older (48 weeks) mice. Interestingly, baseline MAP was significantly increased in mature ACE2^−/y^ (108±2 mmHg, *P*<0.05; Figure 4A), but not in mature NT (102±2 mmHg; Figure 4A), compared to young mice (Figure 2B). Moreover, while cardiac dysautonomia was not further altered by age (Figure 4B,C), vascular sympathetic drive was enhanced in mature ACE2^−/y^, but not in mature NT (Figure 4D). Likewise, oxidative stress was exacerbated in several brain regions of mature ACE2^−/y^ compared to NT mice (*P*<0.05; Figure 4E). Together, these data suggest that ACE2 gene deletion increased the sensitivity to age-dependent oxidative stress and autonomic dysfunction.

### ACE2 gene therapy prevents AngII-mediated oxidative stress and autonomic dysfunction

**Figure 5:**
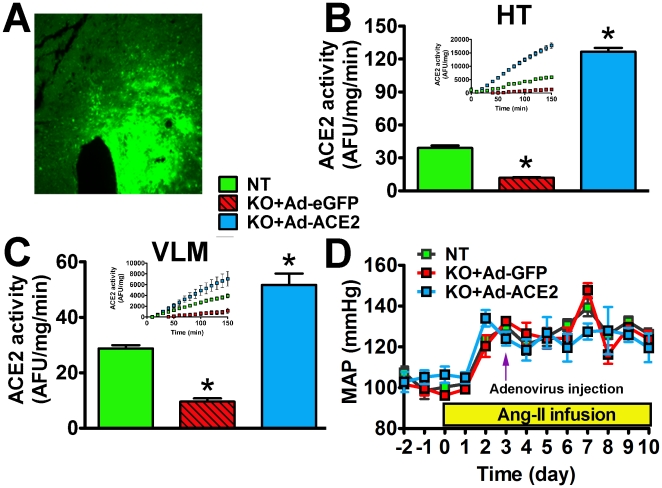
ACE2 expression, activity and MAP in ACE2^−/y^ mice following ACE2 gene therapy to the PVN. Immunohistochemistry (A) showing green fluorescent protein (eGFP) expression targeted to the PVN, 7 days after unilateral administration of adenovirus. ACE2 activity in HT (B) and VLM (C) was significantly lower in ACE2^−/y^ than NT mice (*P<0.01, n = 4). Seven days after ACE2 bilateral adenovirus injection to the PVN, ACE2 activity was significantly increased in HT and downstream VLM in KO mice (*P<0.01 vs. NT, n = 4). Ad-hACE2 injected in the PVN of ACE2^−/y^ on the 3^rd^ day of AngII infusion did not alter the course of the developing hypertension (D, *P*>0.05, n = 8).

**Figure 6:**
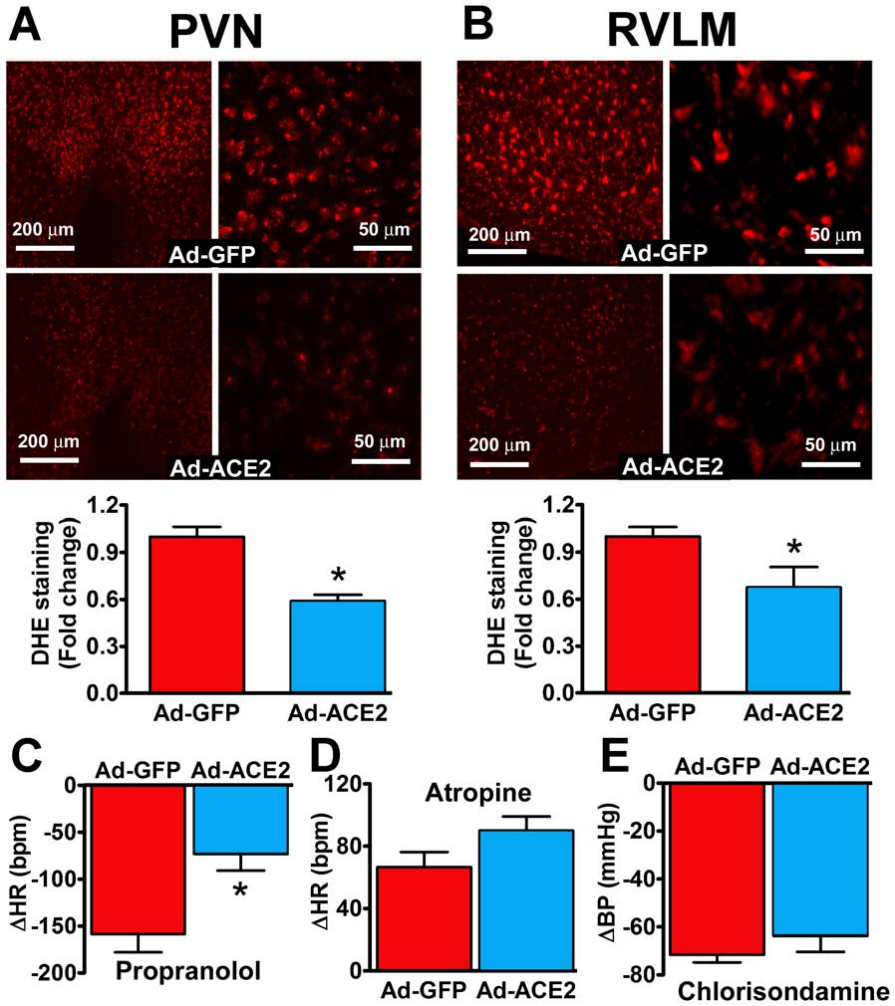
ACE2 gene therapy decreases AngII-mediated oxidative stress and improves autonomic function in ACE2^−/y^ mice. Representative DHE stainings and quantified data showing that AngII-mediated superoxide formation in the PVN (A) and RVLM (B) of ACE2^−/y^ mice (left: 10×, right: 40× magnification) was blunted by Ad-ACE2 infection in the PVN (*P<0.05 vs. Ad-GFP, n = 8). ACE2 over-expression in the PVN improved autonomic function in ACE2^−/y^ mice, as illustrated by a reduced sympathetic drive (C; *P<0.05 vs. Ad-GFP, n = 8) and a trend to increase vagal tone (D; *P*>0.05 vs. Ad-GFP, n = 8). Ad-ACE2 treatment did not change the unaltered vascular sympathetic tone in these mice (E).

Our data indicate that ACE2^−/y^ mice exhibit age-dependent increases in oxidative stress and dysautonomia. Because of the difficulty to work with older animals and since young ACE2^−/y^ infused with AngII have a similar phenotype than mature ACE2^−/y^ mice, we chose to investigate whether ACE2 gene therapy in the brain would prevent autonomic dysfunction and oxidative stress in young ACE2^−/y^ mice infused with AngII. Accordingly, we bilaterally infected these mice with Ad-ACE2 or Ad-GFP in the PVN during AngII infusion. Figure 5A shows typical GFP expression following unilateral targeting of the PVN. Pilot data showed that PVN bilateral injection significantly increased ACE2 activity in both HT and VLM of ACE2^−/y^ mice (Figure 5B,C). Ad-hACE2 injected in the PVN on the 3^rd^ day of AngII infusion did not alter the course of the developing hypertension (Figure 5D). However, DHE staining shows that AngII-mediated ROS production in PVN (0.59±0.04 vs. 1.00±0.06 AU), RVLM (0.67±0.13 vs. 1.00±0.06 AU, *P*<0.05; Figure 6A,B), SFO and NTS (data not shown) of ACE2^−/y^ mice was significantly reduced by ACE2 overexpression. In addition, ACE2 gene therapy reversed the cardiac autonomic dysfunction previously observed in AngII-infused ACE2^−/y^ mice (Figure 2C,D). This was illustrated by a reduction in cardiac sympathetic drive (ΔHR to propranolol: −73±17 vs. −158±19 bpm, *P*<0.05; Figure 6C) and a trend to increase vagal tone (ΔHR to atropine: +90±9 vs. +67±10 bpm, *P* = 0.08; Figure 6D) in AngII-infused ACE2^−/y^ mice. Ad-ACE2 treatment did not change the unaltered vascular sympathetic tone in these mice. These data suggest that reduction of ROS levels in the PVN and RVLM, following ACE2 overexpression, is associated with a reversal of the progressing dysautonomia in ACE2^−/y^ mice.

### ACE2 overexpression decreased NAD(P)H oxidase activity in the brain

**Figure 7:**
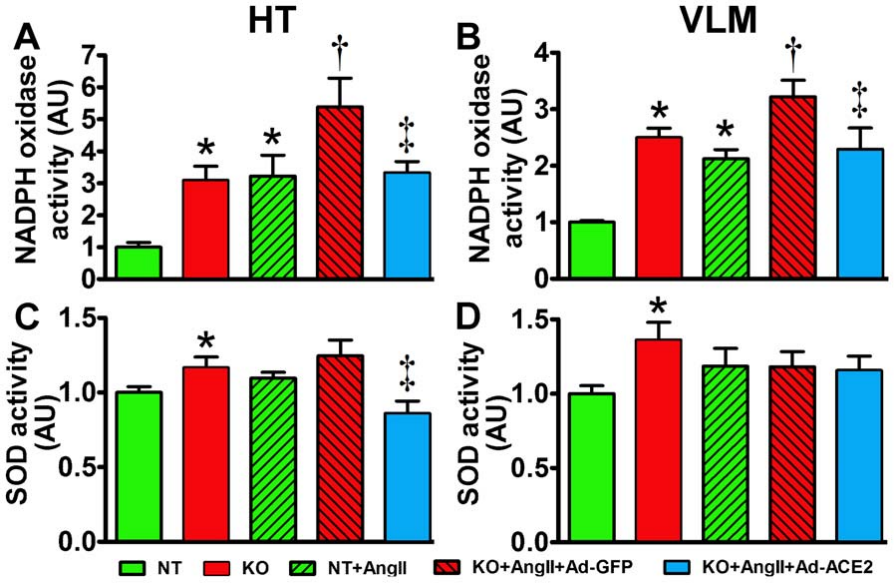
ACE2 overexpression in the PVN decreases NADPH oxidase activity in ACE2^−/y^ mice. NADPH oxidase activity in HT (A) and VLM (B) was significantly higher in ACE2^−/y^ mice on baseline (*P<0.05 vs. NT, n = 4). This enzyme activity was significantly increased in NT following AngII infusion (*P<0.05 vs. NT, n = 4) and remained more elevated in ACE2^−/y^ (†*P*<0.05 vs. NT+AngII). Ad-ACE2 expression in the PVN of ACE2^−/y^ significantly decreased NADPH oxidase activity (‡*P*<0.05 vs. KO+AngII+Ad-GFP). Baseline SOD activity was significantly higher in ACE2^−/y^ mice (C,D; *P<0.05 vs. NT, n = 4). AngII infusion did not change SOD activity in either genotype (*P*>0.05, n = 4). Ad-ACE2 expression in the PVN of ACE2^−/y^ decreased SOD activity in HT (C; ‡*P*<0.05 vs. KO+AngII+Ad-GFP) without changing its activity in VLM (D; *P*>0.05, n = 4).

To investigate the mechanisms involved in ACE2 deletion-mediated oxidative stress in the brain, we assessed NADPH oxidase and superoxide dismutase (SOD) activities in HT and VLM. In ACE2^−/y^, NADPH oxidase activity was significantly increased in both regions compared to NT mice (HT: 3.09±0.44 vs. 1.0±0.14 AU, VLM: 2.50±0.16 vs. 1.0±0.13 AU, *P*<0.05; Figure 7A, B). It remained higher following AngII infusion, although AngII increased NADPH oxidase in both genotypes (*P*<0.05; Figure 7A, B). ACE2 expression in the PVN blunted the AngII-mediated increase in NADPH oxidase activity in ACE2^−/y^ mice (HT: 3.33±0.35 vs. 5.39±0.9 AU, VLM: 2.29±0.38 vs. 3.22±0.29 AU, *P*<0.05; Figure 5A,B) to the level observed in NT+AngII. SOD activity was significantly higher in both brain regions in ACE2^−/y^ compared to NT mice (HT: 1.16±0.07 vs. 1.0±0.03 AU, VLM: 1.36±0.12 vs. 1.0±0.05 AU, *P*<0.05; Figure 7C,D) and was not significantly modified by AngII infusion (Figure 7C,D). Surprisingly, ACE2 overexpression in the PVN of ACE2^−/y^ led to a decreased SOD activity in the HT (0.86±0.05 vs. 1.25±0.1 AU, *P*<0.05 vs. KO+AngII+Ad-GFP; Figure 7C) but not in the VLM (Figure 7D). Altogether, these data suggest that ACE2 gene therapy to the PVN prevents the AngII-mediated increase in NADPH oxidase activity in the HT and downstream VLM, while SOD activity was only affected in HT.

## Discussion

ACE2 is a carboxypeptidase implicated in the conversion of AngII into Ang-(1–7) [25]. It has been well established that AngII stimulates ROS production in both the periphery and CNS; however, the role of ACE2 in oxidative stress, especially in the brain, has not been investigated. The present study highlights the anti-oxidant potential of ACE2 activation in the brain. The main new findings of this study are that ACE2 deletion, age-dependently increases ROS levels, autonomic dysfunction and BP, and exaggerates AngII infusion-induced oxidative stress in brain regions related to cardiovascular function. ACE2 gene therapy reduces AngII-mediated ROS production in the PVN and downstream nuclei associated with inhibition of NADPH oxidase and restoration of autonomic function.

It is well known that blood borne AngII can reach the brain (e.g. via the circumventricular organs) and interact with local AT1R to increase sympathetic drive, ultimately leading to the development of hypertension [26]. We previously showed that brain ACE2 activity was inhibited in a chronically hypertensive mouse model with high AngII levels and that this decrease was mediated by AT1R [16]. Whether the inhibition of ACE2 activity in that model contributes to AngII-mediated oxidative stress is unknown. It has been shown that AngII stimulation of AT1R induces superoxide production in the brain [4], [10], [27] and reduces ACE2 expression at the periphery [28], [29]. In this study, we first tested the interaction between AngII, ACE2, AT1R and AT2R and the participation of oxidative stress in Neuro2A cells. We observed that AngII treatment decreases ACE2 and increased AT1R protein expression. In addition, increase of ACE2 expression by adenovirus infection (∼100-fold) [14] or blockade of AT1R using losartan reduces the AngII-mediated ROS formation. These data suggest that AngII mediates oxidative stress by acting on AT1R. In addition, inhibition of ACE2 and up-regulation of AT1R could also contribute to the increase in ROS. Our data also support an “anti-oxidant” effect for ACE2 activation in neurons. However, ACE2 infection did not completely prevent the AngII-induced ROS production in cells, suggesting a partial metabolism of AngII, most likely due to AngII higher affinity for the AT1R. The ACE2-mediated reduction of ROS formation could result from AngII cleavage, and/or inhibition of AT1R, as we previously showed that over-expression of ACE2 in the brain decreases AT1R expression [14], [18]. Moreover, ACE2 modulation of AngII-mediated oxidative stress could involve the formation of Ang-(1–7). This is supported by observations that Ang-(1–7) is increased in response to ACE2 overexpression [18], stimulates both eNOS and nNOS activities as well as NO release [30], [31], [32], [33], inhibits NAD(P)H oxidase activity [34] and prevents AngII-stimulated superoxide production [35].

At first glance, observations that ACE2 promotes anti-oxidant effects in neurons might seem to diverge from our *in vivo* study. Indeed, ACE2 gene deletion did not change ROS levels in young mice brain and BP in young ACE2^−/y^ mice remained unaffected, consistent with unaltered AngII levels in both plasma and brain. These findings are in line with previous data in similarly engineered ACE2^−/y^ mice, showing no alteration of BP [36], [37], although a small genetic background-associated elevation of baseline BP has also been reported [22]. We speculate that the young ACE2^−/y^ mice are in the early phase of autonomic dysfunction since only cardiac sympathetic and vagal tones are affected at this age and later extended to vascular sympathetic tone, leading to high BP. Indeed, ACE2 removal resulted in age-dependent increase in brain ROS, progressive autonomic dysfunction and higher BP in ACE2^−/y^ mice. On the other hand, ACE2 gene therapy, in AngII-infused mice, prevented increased ROS levels, cardiac sympathetic drive and impaired vagal tone, suggesting that the carboxypeptidase can lessen oxidative stress and restore autonomic function. Unlike previous reports from our group and others [14], [17], ACE2 gene therapy to the brain did not alter the course of AngII-mediated hypertension. This is likely due to the virus being delivered 3 days after the beginning of AngII infusion. Indeed, it would have taken an additional 3 days for the adenovirus to express hACE2 [14], making it possible to see local effects on ROS and autonomic function but more difficult to see a correction of BP level that is also affected by peripheral factors such as vasculature and kidney function.

As also shown by Gurley et al. in plasma [22], AngII infusion resulted in elevated systemic and brain AngII levels in ACE2^−/y^, contributing to the worsening of autonomic dysfunction in these mice. In addition, increased AngII levels also contributed to a higher ROS production in ACE2^−/y^ mice, confirming the pivotal role of the enzyme in AngII metabolism and prevention of AngII-mediated oxidative stress. Altogether, these observations suggest that the lack of ACE2 activity leads to a progressive rise in ROS levels that might contribute to impaired cardiac sympathetic drive, vagal tone and ultimately lead to generalized autonomic dysfunction and high BP.

PVN and RVLM are important brain regions controlling autonomic function [38], and increased ROS formation in these nuclei mediates sympathoexcitation [8], [39]. The observation that ACE2 over-expression in the PVN prevents AngII-mediated ROS production in this region and the downstream RVLM, highlights the importance of direct connections between these 2 regions [40]. More importantly, the improvement of autonomic function in ACE2^−/y^ mice following ACE2 gene therapy in the PVN indicates the ability of central ACE2 in countering oxidative stress not only locally but also in distant regions of the brain. One possible explanation is the ability of ACE2 to be shed from the plasma membrane and released in the surrounding milieu [14], here the cerebrospinal fluid, thus increasing enzyme activity far-away from the delivery site, such as the VLM. This could explain the lower ACE2 activity levels in the VLM versus HT, following virus injection. This hypothesis is also consistent with similar changes in ROS levels in SFO and NTS (data not shown) which may have contributed to the overall restoration of autonomic function.

NAD(P)H oxidase catalyzes the reduction of molecular oxygen to form superoxide. It can be activated by AngII, endothelin, PDGF, TNFα and others [41]. AngII induces ROS formation mainly by NAD(P)H oxidase activation [3]. Interestingly, the loss of ACE2 in our mice increased baseline NADPH oxidase activity in the HT and VLM despite any obvious change in brain or plasma AngII levels, suggesting that additional factors are involved in ACE2 deletion-mediated activation of NADPH oxidase. Increased baseline NADPH oxidase activity in young ACE2^−/y^ did not result in apparent ROS formation in these regions, while a general increase in oxidative stress was observed in mature mice. This suggests that increased NADPH oxidase activity may have led to a progressive accumulation of ROS in various brain regions related to the regulation of cardiovascular function which in turn could contribute to autonomic dysfunction and elevated BP in ACE2^−/y^ mice. The increase in NADPH oxidase activity was associated with parallel elevation of SOD activity, suggesting that a balance between ROS generation (NADPH oxidase) and oxidant scavenging (SOD) in HT and VLM could explain the unaltered baseline ROS levels in the young ACE2^−/y^ mice. ACE2 removal exaggerated AngII infusion-induced NAD(P)H oxidase activation, while it was not able to further increase SOD activity, therefore resulting in an unbalance between production and scavenging of ROS and leading to augmented ROS levels. ACE2 overexpression in the PVN reduced AngII-dependent NADPH oxidase activation in the ACE2^−/y^ mice HT and VLM, suggesting that the beneficial effects of central ACE2 in modulating oxidative stress, involve NADPH oxidase inhibition. Surprisingly, this reduction was accompanied by a decrease in SOD activity in the HT but not the VLM, supporting the idea that ACE2 overexpression in the PVN may have affected the oxidant scavenging enzymes in this area. More studies are needed to clarify this phenomenon.

In addition to ROS, target of rapamycin (TOR) is involved in aging. It has been shown that activation of TOR pathway increases protein synthesis, stimulates cell mass growth and inhibits translation and autophagy, therefore promoting aging [42], while inhibition of the TOR pathway prolongs life in yeast [43], worm [44] and flies [45]. It has been reported that AngII activates mTOR (TOR in mammals) via PI3-kinase signaling in human coronary smooth muscle cells [46], and ROS can also activate [47] or be activated by mTOR [48]. Although we did not investigate mTOR pathway in this study, the role of mTOR cannot be excluded from our aged ACE2^−/y^. Moreover, ROS-mediated aging could involve activation of various additional pathways such as SirT1 [49], Ask1-signalosome and p38 MSPK [50]. Further studies are needed to address whether these pathways are involved in age-related high blood pressure following ACE2 deletion.

In summary, our data suggest that ACE2 gene deletion age-dependently promotes oxidative stress, while ACE2 gene therapy to the PVN decreases ROS formation via NADPH oxidase inhibition, thus improving autonomic function and preventing the long term increase in BP.

### Limitations of the study

Despite a lack of ACE2, our data failed to show an increase in plasma and brain AngII levels. While plasma AngII levels have been reported to be normal or increased in ACE2^−/y^ mice [51], the lack of increased brain AngII levels could be related to the sensitivity of our approach. Indeed, we previously observed that icv losartan reduces baseline BP in ACE2^−/y^ mice [52], suggesting either increased AngII or AT1R levels in the brain. In the present study, we measured AngII in a brain sample overlapping several nuclei which may have resulted in the dilution of AngII levels.

Another limitation of the study is our choice to perform ACE2 gene therapy in young ACE2^−/y^ mice. This choice was motivated by the difficulty in performing multiple survival surgeries (telemetry probe, osmotic pump implantations, PVN viral injection) combined to multiple drug injections (autonomic function) in older mice. A better approach would have been to inject the ACE2 virus in young mice and monitor the effects on autonomic function and oxidative stress in old mice. However, the limited life (7–10 days) of the ACE2 adenovirus did not permit such experiments.

### Perspectives

Oxidative stress is involved in many diseases. While several effective approaches already exist to prevent ROS formation and end-organ damage, including ACE inhibitors and AngII receptor blockers, ACE2 offers a possible new option to fight oxidative damage by targeting the multiple actors involved in ROS formation. ACE2 gene therapy or drug-mediated increase in ACE2 activity could promote: 1) AngII degradation, thus reducing the activation of the ROS formation cascade; 2) AT1R downregulation, preventing the activation of NAD(P)H oxidase; and 3) Ang-(1–7) formation, further inhibiting the NAD(P)H oxidase complex. While AT1R blockers increase AngII levels, they also provide the opportunity for AT2R to be activated. However, it is still unclear how these receptors could modulate oxidative stress. Future studies will need to address the potential for these mechanisms, as well as the prospect of combined blockade of AT1R and ACE2 activation.
